# WAVE2 Protein Complex Coupled to Membrane and Microtubules

**DOI:** 10.1155/2012/590531

**Published:** 2012-01-18

**Authors:** Kazuhide Takahashi

**Affiliations:** Molecular Cell Biology Division, Kanagawa Cancer Center Research Institute, Yokohama 241-0815, Japan

## Abstract

E-cadherin is one of the key molecules in the formation of cell-cell adhesion and interacts intracellularly with a group of proteins collectively named catenins, through which the E-cadherin-catenin complex is anchored to actin-based cytoskeletal components. Although cell-cell adhesion is often disrupted in cancer cells by either genetic or epigenetic alterations in cell adhesion molecules, disruption of cell-cell adhesion alone seems to be insufficient for the induction of cancer cell migration and invasion. A small GTP-binding protein, Rac1, induces the specific cellular protrusions lamellipodia via WAVE2, a member of WASP/WAVE family of the actin cytoskeletal regulatory proteins. Biochemical and pharmacological investigations have revealed that WAVE2 interacts with many proteins that regulate microtubule growth, actin assembly, and membrane targeting of proteins, all of which are necessary for directional cell migration through lamellipodia formation. These findings might have important implications for the development of effective therapeutic agents against cancer cell migration and invasion.

## 1. Introduction

The cell adhesion molecule E-cadherin is among the key molecules in the formation of the epithelial junctional complex [1–3]. E-cadherin forms homodimers in the extracellular domain between adjacent cells [4] and interacts intracellularly with a group of proteins collectively named catenins [5, 6]. Both the cadherin cytoplasmic domain and the associated catenins are required for full cell adhesion [7, 8], and *α*-catenin is suggested to play a role in anchoring the cadherin-catenin complex to actin-based cytoskeletal components that include *α*-actinin and vinculin [9]. Loss or significant reduction of E-cadherin expression has been observed in many epithelial cancers [10–14], and the *α*-catenin gene is occasionally lost [14, 15] or mutated in human cancer cells lines [16]. Unlike E-cadherin and *α*-catenin, *β*-catenin loses its function upon tyrosine phosphorylation in response to growth factors [17–20] or v-Src [21, 22]. Tyrosine phosphorylation of *β*-catenin results in loss of anchoring of the E-cadherin-catenin complex to the network of actin filaments (F-actin) [23, 24]. In normal epithelial cells in culture, tyrosine-phosphorylated *β*-catenin needs to be dephosphorylated in order to link the E-cadherin-catenin complex to F-actin. In fact, several protein tyrosine phosphatases (PTPs), including DEP-1, PTP1B, and PTP*μ*, are upregulated with increasing cell density [25, 26], and they associate with the E-cadherin-catenin complex [27–31]. Therefore, constitutive tyrosine phosphorylation of *β*-catenin and concomitant loss of PTP might cause loss of E-cadherin-mediated cell-cell adhesion in cancer cells.

 Another mode of dysfunction of cell-cell adhesion is induced by perturbation of F-actin assembly to which the E-cadherin-catenin complex anchors. IQGAP1, the IQ moti-containing guanine nucleotide-activating protein 1 (IQGAP1) [32], is an actin cross-linking protein [33, 34] or scaffold protein [35]. IQGAP1 is recruited by protein phosphatase (PP) 2A to the E-cadherin-catenin complex that is constitutively associated with the small GTP-binding protein Rac1 [36, 37], thereby leading to rearrangement of F-actin by IQGAP1 in corporation with Rac1 to which the E-cadherin-catenin complex anchors. Because inhibition of PP2A activity or loss of PP2A expression results in E-cadherin endocytosis [37, 38], PP2A is considered to be involved in the establishment and maintenance of E-cadherin-mediated cell-cell adhesion as well as in a wide variety of biological processes such as tumor suppression [39–41], formation of tight junctions [42], and integrin-mediated cell-substratum adhesion [43–48]. Loss of cell-cell adhesion due to the internalization or endocytosis of E-cadherin without significant alterations in E-cadherin expression has been observed in many cells [38, 49–52]. Despite this, PP2A expression is lost in both invasive and noninvasive cancer cells [38]. This implies that the loss of cell-cell adhesion is necessary but insufficient alone for cell migration and invasion.

 In this paper, we focus on recent advances in the understanding of the mechanisms underlying regulation of cell migration and invasion that have been possible through the use of biochemical and pharmacological approaches.

## 2. Regulation of Cell Migration

Cell migration and invasion generally require rearrangement of F-actin at the leading edge of cells [53] and are associated with the formation of specific cellular protrusions termed lamellipodia or filopodia [54]. Rearrangement of F-actin is directed by the Arp2/3 complex through induction of nucleation and branching of F-actin [54–56] and is regulated by small GTP-binding proteins such as Rac1 and Cdc42 [57] through Wiskott-Aldrich syndrome protein (WASP) and WASP verprolin homology proteins (WAVE) [55, 58–62]. N-WASP is a member of the WASP family of proteins [58, 60] and is thought to be necessary for lamellipodia formation [63]. WAVE family proteins, including WAVE1, -2, and -3 [58, 61], induce both lamellipodia [64–66] and filopodia [67]. Among the WASP/WAVE family, WAVE2 has been the most intensively investigated and has been identified as functioning downstream of Rac [68, 69].

**Figure 1:**
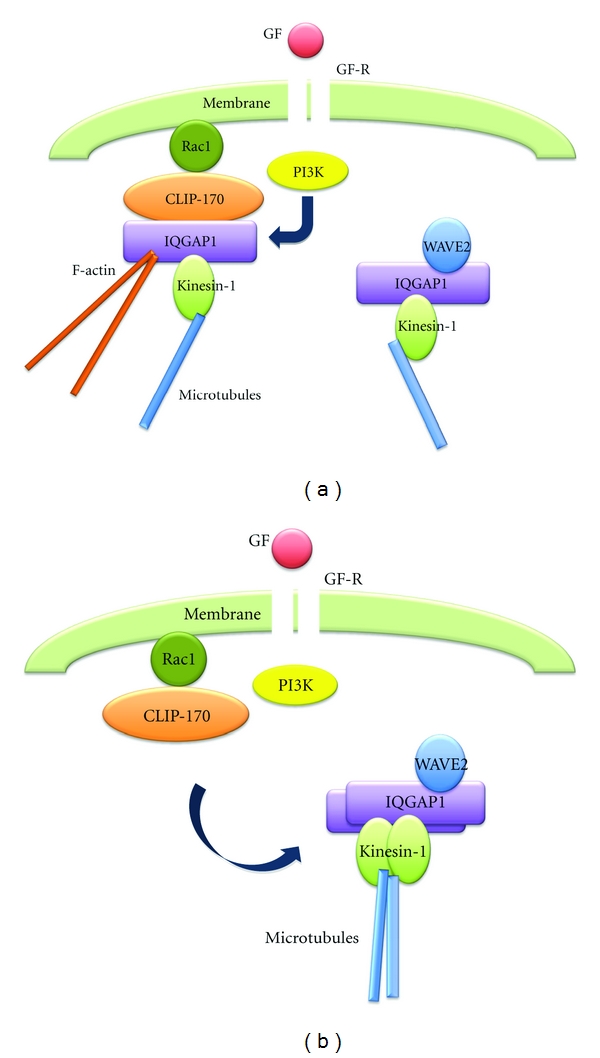
PI3K-dependent dissociation of IQGAP1 and kinesin-1 from the Rac1-CLIP-170 complex and successive binding of them to WAVE2 after GF stimulation. (a) IQGAP1 and kinesin-1, which are bound to Rac1 via CLIP-170 in growth-arrested cells, dissociate from the Rac1-CLIP-170 complex after GF stimulation in a PI3K-dependent manner. (b) Dissociated IQGAP1 and kinesin-1 bind to the WAVE2 complex, which consists of IQGAP1 and kinesin-1. GF: growth factor; GF-R: growth factor receptor; PI3K: phosphoinositide 3-kinase; CLIP-170: cytoplasmic linker protein 170; IQGAP1: IQ motif-containing guanine nucleotide activating protein 1; WAVE2: Wiskott-Aldrich protein verprolin homology protein 2; F-actin: actin filaments.

Although direct interaction between Rac1 and WAVE2 is not detected in human breast cancer cells, Rac1 forms a complex with CLIP-170, a microtubule-binding protein [70, 71], an actin cross-linking protein IQGAP1 [33, 34], and kinesin-1, one of the major motor proteins [72, 73], under the growth-arrested conditions [74] (Figure 1(a)). CLIP-170 and IQGAP1 bind microtubules and F-actin, respectively, and kinesin-1 transports many cytoplasmic vesicles, proteins, and mRNAs as “cargo” toward the growing plus-ends of microtubules. Therefore, formation of the Rac1, CLIP-170, IQGAP1, and kinesin-1 complex induces linking of Rac1 to both F-actin and microtubules by IQGAP1 and CLIP-170, respectively [74, 75]. Depletion of CLIP-170 expression causes growth factor-independent dissociation of IQGAP1 and kinesin-1 from Rac1 and the promotion of random lamellipodia formation and invasion [74]. This suggests that CLIP-170 may play a role in preventing cells from growth factor-independent lamellipodia formation and invasion, by tethering IQGAP1 and kinesin-1 to Rac1 until stimulation by growth factors. Following the stimulation of cells with growth factor, IQGAP1 and kinesin-1 dissociate from the Rac1-CLIP-170 complex [74] (Figure 1(b)). As lamellipodia formation and dissociation of IQGAP1 and kinesin-1 are inhibited by a phosphoinositide 3-kinase (PI3K) inhibitor, the dissociation of IQGAP1 and kinesin-1 from the Rac1-CLIP-170 complex is a prerequisite for lamellipodia formation in response to growth factor stimulation; this depends on PI3K that is activated by the activated growth factor receptor (Figure 1(a)).

## 3. Membrane Targeting of WAVE2 along Microtubules

**Figure 2:**
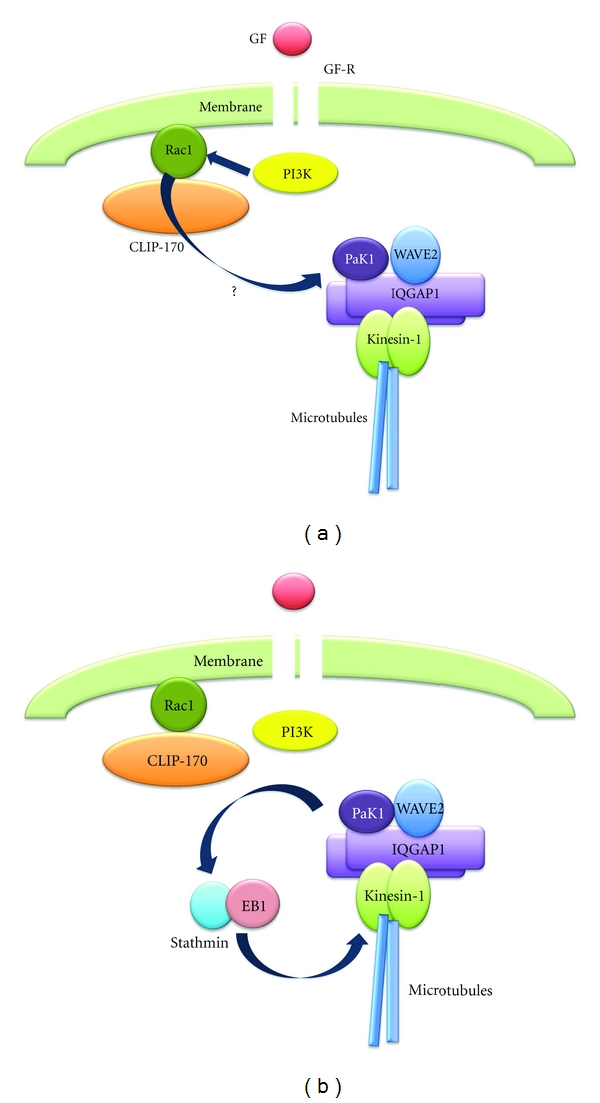
Activation of WAVE2-bound Pak1, phosphorylation of stathmin by Pak1, and recruitment of the phosphorylated stathmin-EB1 complex to the microtubule ends that bear the WAVE2 complex. (a) WAVE2-bound Pak1 is activated and in turn phosphorylates stathmin that complexes with EB1. (b) The phosphorylated stathmin-EB1 complex is recruited to microtubule ends that bear the WAVE2 complex through kinesin-1. Pak1: p21-activated protein kinase 1; EB1: microtubule end-binding protein 1.

In order to form lamellipodia, WAVE2 needs to be transported to the leading edge of cells prior to lamellipodia formation [76, 77]. WAVE2 is known to form multiprotein complexes that include Abi-1, Sra-1, Nap-1, and HSPC300 [78–80]. WAVE2 also forms a complex with IQGAP1 and kinesin-1 in growth-arrested breast cancer cells (Figure 1(a)), and the amounts of these proteins relative to WAVE2 increase after stimulation of the cells with growth factor [65] (Figure 1(b)). As IQGAP1 and kinesin-1 dissociate from the Rac1-CLIP-170 complex upon growth factor stimulation, without significant alterations in the total amounts of IQGAP1 and kinesin-1, IQGAP1 and kinesin-1 that are bound to WAVE2 might be proteins that previously dissociated from the complex (Figure 1(b)). In spite of this, additional binding of IQGAP1 and kinesin-1 to WAVE2 appears to be insufficient for WAVE2 translocation to the cell cortex. Many investigations have revealed that a p21-activated protein kinase Pak1 [81, 82], which is one of the downstream effectors of Rac1 [81, 83, 84], is constitutively associated with WAVE2 [66] (Figure 2(a)). Pak1 is thought to regulate not only actin reorganization through several reported substrates, including LIM kinase [85], p41-Arc [86], and filamin [87], but also microtubule dynamics through stathmin [88–90], a microtubule destabilizing protein [91, 92]. As direct interaction between Rac1 and Pak1 or WAVE2 is not detected in breast cancer cells, it is reasonable to assume that *β*PIX, the Pak-interacting nucleotide exchange factor of Rac/Cdc42 [93], plays an important role in the signal transduction between Rac1 and Pak1. *β*PIX is constitutively coupled to GIT1 and recruited to WAVE2-bound Pak1 in response to growth factor stimulation [94]. Upon growth factor stimulation, the WAVE2-bound Pak1 is activated and in turn phosphorylates stathmin [66] (Figures 2(a) and 2(b)). The putative phosphorylation sites within stathmin are Ser16, Ser25, Ser38, and Ser63 [95]. Among these, growth factor stimulation causes phosphorylation at Ser25 and Ser38, although phosphorylation at Ser38 alone is dependent on Pak1 in breast cancer cells [66]. This implies that serine/threonine protein kinases other than Pak1 phosphorylate stathmin at Ser25. In either case, the microtubule destabilizing activity of stathmin is inactivated by phosphorylation [88–90], and the phosphorylated stathmin is recruited to kinesin-1; this complexes with WAVE2 and Pak1 and leads to the promotion of microtubule growth [66] (Figure 2(b)). Stathmin binding to kinesin-1 was thought to be transient and to play a role in the transportation of tubulin heterodimers to the microtubule ends [95]; however, stathmin binding to kinesin-1 and tubulin heterodimers is maintained after growth factor stimulation [66]. This suggests the presence of a partner protein that facilitates the sustained linking of stathmin to the microtubule ends. The dynamic properties of microtubules are regulated by “plus-end-binding proteins” [96–98] that include CLIP, CLIP-associated protein (CLASPs), dynein/dynactin, APC (adenomatous polyposis coli), and EB-family proteins [99–101]. EB1 recognizes specific sites at the ends of growing microtubules [102] and promotes persistent microtubule growth [103]. Investigation of the stathmin-binding partners revealed that stathmin is constitutively associated with EB1 in the cytoplasm of growth-arrested cells [104]. After phosphorylation of stathmin by Pak1, the phosphorylated stathmin-EB1 complex becomes associated, in an EB1-dependent manner, with the ends of microtubules that bear the WAVE2 complex [104]. Depletion of stathmin does not inhibit microtubule growth but inhibits WAVE2 translocation and lamellipodia formation [66], while EB1 depletion does not inhibit microtubule growth but inhibits stathmin binding to kinesin-1 at the microtubule ends, WAVE2 translocation, and lamellipodia formation [104]. Taken together, growth factor-induced and Pak1-dependent phosphorylation of stathmin triggers the EB1-mediated specific binding of the phosphorylated stathmin-EB1 complex to the WAVE2-bearing microtubule ends, thereby leading to WAVE2 translocation and lamellipodia formation (Figure 2(b)).

## 4. Control of Directional Lamellipodia Formation toward Growth Factor

**Figure 3:**
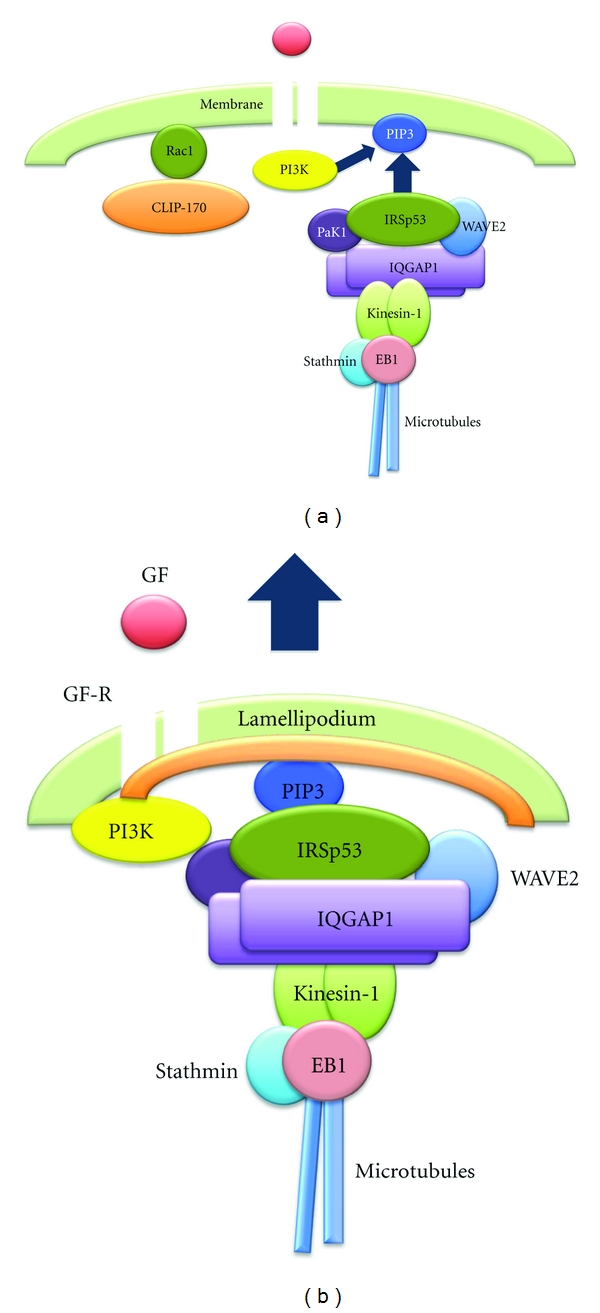
Directional membrane targeting and anchoring of WAVE2 to PIP_3_ by IRSp53, leading to the directional formation of lamellipodia toward GF. (a) Promotion of microtubule growth through recruitment of the stathmin-EB1 complex to the microtubule ends induces membrane targeting of the WAVE2 complex along microtubules. The WAVE2 complex targets the cell cortex and is anchored by IRSp53. IRSp53 links WAVE2 to PIP_3_ produced by PI3K near GF-R locally activated in the membrane region facing GF. (b) Colocalization of WAVE2, IRSp53, and PIP_3_ results in the directional formation of lamellipodia towards high concentrations of GF. IRSp53: insulin receptor substrate p53; PIP_3_: phosphoinositide 3,4,5-triphosphate.

The process of cancer cell migration and invasion involves intravasation and extravasation of cells into and out of blood vessels [105]. Therefore, it is highly likely that cancer cell migration and invasion are directionally controlled by extracellular stimuli such as serum growth factors. If this theory is correct, promotion of persistent growth of the WAVE2-bearing microtubules by the binding of the stathmin-EB1 complex seems to be insufficient for the directional membrane anchoring of microtubules, that is, without the aid of partner proteins. For example, the end-binding proteins CLASPs link microtubule ends to the cell cortex by the phosphatidylinosito 3,4,5-triphosphat- (PIP_3_-) binding protein LL5*β* as a partner protein [106]. PIP_3_, a lipid component of the cytoplasmic membrane produced by PI3K, is known to be the membrane target of many proteins [107–111] that contain the pleckstrin homology (PH) domain (e.g., PKB/Akt [112, 113]), the basic region (e.g., WASP and WAVE2 [76, 114]), or the IRSp53/missing-in-metastasis (MIM) domain IMD (e.g., IRSp53 [115, 116]). In lamellipodia-forming breast cancer cells, IRSp53 is constitutively associated with the WAVE2 complex that involves EB1 and plays an indispensable role in anchoring the complex to PIP_3_ [117]. IRSp53 is a linker protein [118–120] that interacts with the proline-rich region of WAVE2 through its central Src-homology 3 (SH3) domain [118, 121]. Therefore, directional cell migration through lamellipodia formation may be regulated by the interaction between WAVE2-bound IRSp53 as the PIP_3_-binding partner protein and PIP_3_ as the membrane target molecule; this suggests that the direction of membrane anchoring of microtubules that bear the WAVE2 complex is determined by the sites where PIP_3_ is produced. In cells cultured in a chemotaxis chamber, only growth factor receptor in the membrane region facing high concentrations of growth factor was activated [104]. Local activation of the receptor causes recruitment of PI3K that in turn produces PIP_3_ in close proximity to the activated receptor [104] (Figure 3(a)), thereby leading to local interaction between IRSp53-bound WAVE2 and PIP_3_, and thus the directional formation of lamellipodia towards the growth factor source (Figure 3(b)). 

**Figure 4:**
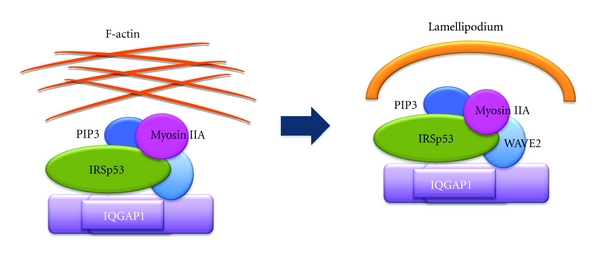
The role of nonmuscle myosin IIA in lamellipodia formation. The network of F-actin, initially rearranged by WAVE2, IRSp53, and PIP_3_, is assembled into lamellipodium, a convex arc of F-actin, by the WAVE2-bound nonmuscle myosin IIA heavy chain MYH9 and the motor activity of nonmuscle myosin II.

 In the context of PIP_3_-binding proteins for lamellipodia formation, nonmuscle myosin IIA heavy chain MYH9 [122–124] has the ability to bind to both PIP_3_ and WAVE2 [125]. Whereas MYH9 binding to WAVE2 is constitutive and requires the motor activity of myosin II, MYH9 binding to PIP_3_ is induced only after WAVE2 membrane targeting by growth factor activation [125]. The binding of MYH9 to WAVE2 is probably through the interaction between the SH3-like domain of MYH9 and the proline-rich domain of WAVE2; however, MYH9 lacks a PH domain, unlike myosin 1b and myosin X, which bind to PIP_3_ through their PH domains [126, 127]. Nevertheless, both MYH9 and the motor activity of nonmuscle myosin II are crucial for lamellipodia formation, as they induce convex F-actin arcs at the leading edge of cells [125] (Figure 4). Nonmuscle cells express multiple myosin II proteins, including myosin IIA, myosin IIB, and myosin IIC [122–124]; these are implicated in regulating many cellular processes, including cell spreading, migration, and cytokinesis [128–131], through generating the intracellular contractile forces and tension as conventional motor proteins by associating with F-actin. Thus, myosin IIA might also play a crucial role in cell migration through lamellipodia formation by providing the contractile forces and tension for the F-actin network initially rearranged by WAVE2, IRSp53, and PIP_3_ through the variable turnover dynamics of F-actin [132, 133] to form a convex arc at the leading edge of cells (Figure 4). As IRSp53 exhibits F-actin-bundling activity [134, 135], the formation of a lining of F-actin at the leading edge of cells might be mediated by nonmuscle myosin IIA in cooperation with IRSp53.

 Among the proteins and molecules that are involved in regulation of lamellipodia formation, WAVE2, N-WASP, PI3K, Rac1, stathmin, and microtubules are also necessary for cell invasion [69, 136–139]. Because cell invasion is accomplished by intensive accumulation of the F-actin bundles at the tips of cell protrusions where cells invade through the basement membrane matrix and narrow gaps [138], the signaling and regulatory molecules leading to cell invasion may share many common molecules, leading to cell migration through lamellipodia formation.

## 5. Conclusion

Cell migration and invasion are believed to require the formation of lamellipodia at the leading edge of cells by rearrangement of F-actin. Lamellipodia formation is preceded by membrane targeting of WAVE2 along microtubules. WAVE2 forms multiprotein complexes consisting of IQGAP1, kinesin-1, Pak1, IRSp53, and nonmuscle myosin IIA. Membrane targeting of the WAVE2 complex is triggered by binding of IQGAP1 and kinesin-1, which are dissociated from the Rac1-CLIP-170 complex upon PI3K activation by the activated growth factor receptor. Concomitantly, WAVE2-bound Pak1 after Rac1-dependent activation phosphorylates stathmin, followed by binding of the stathmin-EB1 complex to the microtubule ends that bear the WAVE2 complex; this results in the promotion of persistent microtubule growth towards the cell cortex. The WAVE2 complex that targets the cell membrane is anchored by WAVE2-bound IRSp53 and nonmuscle myosin IIA to PIP_3_; PIP_3_ is produced by PI3K near the growth factor receptor that is locally activated in the membrane region facing higher concentrations of growth factor. Colocalization of WAVE2, IRSp53, nonmuscle myosin IIA, and PIP_3_ induces the directional formation of a convex F-actin arc lamellipodium. The discovery of many essential signaling and regulatory molecules for cell migration might have important implications for the development of effective therapeutic agents against cancer cell migration and invasion.
